# General anesthesia with propofol during oocyte retrieval and in vitro fertilization outcomes: retrospective cohort study

**DOI:** 10.1038/s41598-023-35224-2

**Published:** 2023-05-17

**Authors:** Einat Haikin Herzberger, Omri Levy, Bei Sun, Netanella Miller, Roni Rahav, Elad Dana, Shaul Raviv, Anat Hershko-Klement, Amir Wiser

**Affiliations:** 1 IVF Unit, Department of Obstetrics and Gynecology, Meir Medical Center, Kfar-Saba, Israel; 2 Sackler Faculty of Medicine, Tel Aviv University, Tel Aviv, Israel; 3 Department of Anesthesiology and Critical Care Medicine, Meir Medical Center, Kfar-Saba, Israel; 4 Department of Obstetrics and Gynecology, Hadassah Mt. Scopus medical center, Faculty of medicine, The Hebrew University, Jerusalem, Israel

**Keywords:** Outcomes research, Endocrine reproductive disorders

## Abstract

General anesthesia is frequently administered during oocyte retrieval. Its effects on the outcomes of IVF cycles are uncertain. This study investigated whether administration of general anesthesia (specifically propofol) during oocyte retrieval affects IVF outcomes. A total of 245 women undergoing IVF cycles were included in this retrospective cohort study. IVF outcomes of 129 women who underwent oocyte retrieval under propofol anesthesia and 116 without anesthesia were compared. Data were adjusted for age, BMI, estradiol on triggering day and total gonadotropin dose. The primary outcomes were fertilization, pregnancy and live birth rates. A secondary outcome was the efficiency of follicle retrieval associated with the use of anesthesia. Fertilization rate was lower in retrievals under anesthesia compared to without (53.4% ± 34.8 vs. 63.7% ± 33.6, respectively; *p* = 0.02). There was no significant difference in the ratio of expected to retrieved oocytes between retrievals with and without anesthesia (0.8 ± 0.4 vs. 0.8 ± 0.8, respectively, *p* = 0.96). The differences in pregnancy and live birth rates between the groups were not statistically significant. General anesthesia administered during oocyte retrieval may have adverse effects on the fertilization potential of oocytes. This impact on the developmental potential of oocytes may lead to negative IVF outcomes and should be investigated further.

## Introduction

Oocyte retrieval is an important component of the in vitro fertilization (IVF) cycle. Although minimally invasive, the procedure is painful. Therefore, it is usually performed under anesthesia^1^.

General anesthesia may affect IVF outcomes indirectly through systemic hemodynamic and biochemical changes and directly through its effects on oocytes. Significant decreases in blood pressure have been reported 2 min after induction of anesthesia^2^. Consequently, perfusion to the follicles and the endometrium is decreased. Significant decreases in hemoglobin concentration and plasma cortisol level, and increased blood glucose were observed 30 min after anesthesia induction^3^. Intravenous propofol, with short induction and recovery times, is the drug of choice for oocyte retrieval performed under sedation. Despite its favorable pharmacokinetic properties and safety profile, animal studies have shown that it can affect early embryonic development^4–6^. Propofol can accumulate in the follicular fluid and even brief exposure led to deleterious effects on subsequent cleavage events of mouse oocytes^4^. Another group demonstrated that exposure of metaphase II mouse oocytes to propofol significantly reduced the rate of sperm fusion^5^. Only a few human studies have been reported^7–9^. These results led us to investigate the possible effects of propofol exposure during human oocyte retrieval on the developmental potential of oocytes.

The current study assessed whether general anesthesia with propofol during oocyte retrieval had a negative impact on the fertilization rate of oocytes, the clinical pregnancy rate per embryo transfer (ET) and the live birth rate.

## Methods

The study was performed at a tertiary, university-affiliated medical center from November 2017 through December 2019. November 2017 was chosen as the starting point because the anesthesia department transitioned to an electronic medical record system at that time. Demographics, cycle treatment and anesthesia parameters and outcomes of IVF treatment were collected from patients' electronic medical records.

Patient data from women 18 to 45 years old undergoing a fresh cycle of IVF treatment were retrospectively analyzed. Patients with chronic inflammatory disease, vascular disease or presentation of acute illness during IVF cycle were not included. A total of 245 fresh IVF cycles were included in the analysis, of which 129 oocytes were retrieved under general anesthesia and 116 without anesthesia. Patients were matched for age and date of retrieval.

Trigger injections were administrated when the leading follicle was ≥ 17–18 mm, with adequate estradiol levels. For ovulation triggering, either GnRH-a 0.2 mg decapeptyl (0.1 mg, 105 µg triptorelin acetate, Ferring, Germany) or r-hCG 250 mcg (Ovitrelle, 250 µg Choriogonadotropin alfa, Merck Serono S.A., Switzerland) was used, according to the E2 level and risk for ovarian hyperstimulation syndrome. Oocyte retrieval was performed 36 h after triggering.

Patients treated in our unit were able to choose between undergoing oocyte retrieval with or without anesthesia, according to their preference. Patients undergoing the procedure with anesthesia were evaluated preoperatively by an anesthesiologist. The following anesthesia data were obtained from patients' electronic medical records: duration (min), dosage (mg), peripheral capillary oxygen saturation and mean arterial pressure. All patients undergoing oocyte retrieval with anesthesia fasted for at least 8 h before the procedure. They were monitored using noninvasive arterial pressure, continuous electrocardiogram, and pulse oximetry. The airway was maintained using an oxygen mask only. In rare cases, where patient characteristics suggested a high aspiration risk or when mask ventilation only was inadequate, a laryngeal mask or endotracheal tube was employed. A peripheral venous 20/22-gauge catheter was placed. Fentanyl (100 mcg) was administered for analgesia. Propofol (1–2 mg/kg) was administered for induction, with additional IV boluses according to the duration of the procedure, level of sedation and maintenance of anesthesia.

If hypotension occurred, it was usually transient and resolved spontaneously, or was treated with vasoactive medications such as phenylephrine (100 mcg boluses) or ephedrine (5 mg boluses), at the anesthesiologist’s discretion.

Patients without anesthesia were not treated with analgesic medications during the retrieval. If requested, they received analgesics, mostly intravenous or oral paracetamol (1 g) in the recovery room.

The primary outcomes were fertilization rate, pregnancy rate and live birth rate. Fertilization rate was calculated as the number of normal fertilized oocytes among inseminated or injected oocytes. Pregnancy rate was calculated based on cycles that resulted in ET only. Live birth rate was calculated from cycles that resulted in pregnancy.

A secondary outcome was the efficiency of follicle retrieval associated with the use of anesthesia. This was calculated based on the ratio of expected oocytes to retrieved oocytes. The number of expected oocytes (follicle size ≥ 10) was extracted from sonographic examination records.

Embryos were graded from one to four, based on percentage of fragmentation and cell counts: grade 4, equal-size symmetrical cells with no fragmentation; grade 3, equal-size symmetrical cells with less than 10% fragmentation; grade 2, non-symmetrical blastomeres with 10–50% fragmentation; and grade 1, more than 50% fragmentation. ET was accomplished on day 3 after oocyte collection. Serum β-hCG was measured 12 days after ET. Transvaginal ultrasound was performed 2 weeks later to confirm a viable pregnancy. Clinical pregnancy was defined as the presence of an intrauterine gestational sac with fetal cardiac activity.

### Statistical analysis

Data were analyzed using SPSS v.25.0 for Windows (IBM Corp., Armonk, NY, USA). Descriptive parameters are expressed as mean ± SD. Frequencies are presented as percentages. The student t-test was used to compare continuous parameters and the chi-square test was used to analyze categorical parameters. Linear regression was used to evaluate the effect of the variables on fertilization rate. Pearson and Spearman correlations were used for anesthetic parameters and IVF outcomes. A *P*-value < 0.05 was considered statistically significant. All statistical tests were two-tailed.

### Ethics approval and consent to participate

This study was performed in line with the principles of the Declaration of Helsinki.

The study was approved by the Meir Medical Center Ethics Committee, affiliated with the Sackler faculty of Medicine at Tel Aviv University, Israel. The requirement for informed consent from the study subjects was waived by the IRB Helsinki Committee due to the retrospective nature of the study.

## Results

A total of 245 fresh IVF cycles were included in the analysis, of which 129 oocytes were retrieved under general anesthesia and 116 were retrieved without anesthesia. The main demographic, baseline and treatment characteristics are presented in Table 1. There were no significant differences between patient groups in terms of age, BMI and indications for treatment. Male factor was the indication in 43% of patients undergoing oocyte retrieval with anesthesia versus 41% in patients without anesthesia. Basal FSH was lower for patients undergoing oocyte retrieval with anesthesia compared to patients undergoing retrieval without anesthesia (*p* = 0.02). The total dosage of gonadotropins or triggering agent was similar between the two groups. However, estradiol blood levels and expected number of oocytes were higher in retrievals under anesthesia compared to retrievals without anesthesia (*p* < 0.01 for both). Among women who did not use anesthesia, 53% chose to undergo their next procedure without anesthesia.

**Table 1:** Demographic and treatment data of oocyte retrievals with versus without anesthesia.

Patient Characteristics	Anesthesia (n = 129)	No anesthesia (n = 116)	*p*-value
Age (years)	36.6 ± 4.7	37.6 ± 4.3	0.07
BMI (kg/m^2^)	25.5 ± 6.0	26.1 ± 6.0	0.43
Obesity	28 (21.7%)	25 (21.6%)	0.97
Gravidity	1.2 ± 1.6	1.0 ± 1.3	0.26
Parity	0.6 ± 0.9	0.5 ± 0.6	0.33
Smoking (yes/no)	41 (32.0%)	38 (32.8%)	0.90
Baseline FSH	8.9 ± 4.2	10.4 ± 5.0	0.02
Treatment characteristics			
Gonadotropin dosage (IU/l)	3118.3 ± 1400.3	2809.76 ± 1683.9	0.12
Estradiol levels (picogram/ml)	1255.83 ± 699.1	773.68 ± 666.5	< 0.01
Small follicles expected	6.24 ± 3.2	3.5 ± 3.0	< 0.01
Large follicles expected	2.97 ± 1.8	1.91 ± 1.32	< 0.01
Triggering agent			
r-hCG	115 (89.1%)	111 (95.7%)	0.11
GnRH agonist	8 (6.2%)	4 (3.4%)	
Double	6 (4.7%)	1 (0.9%)	
Intracytoplasmic sperm injection (%)	76 (58.9%)	58 (50.0%)	0.19
Another cycle without anesthesia	32/60 (53%)		

Data are reported as mean ± standard error of the mean or n (%).

Oocyte retrieval and clinical outcomes are shown in Table 2. The total quantity of oocytes retrieved was significantly higher in oocyte retrievals with anesthesia compared to retrievals without anesthesia (*p* < 0.01). There was no significant difference in the percentage of mature oocytes obtained from each group. Fertilization rate was lower in retrievals under anesthesia compared to those without (53.4% ± 34.8 vs. 63.7% ± 33.6 respectively, *p* = 0.02). There was a trend toward lower rate of pregnancies resulting in delivery among retrievals under anesthesia compared to without anesthesia (66.7% vs. 85.0%, *p* = 0.13). Spontaneous abortion occurred in 28.2% of pregnancies with oocytes retrieved under anesthesia compared to 15.0% without anesthesia (*p* = 0.26). The delivery and abortion rates were similar between groups.

**Table 2:** Comparison of cycle outcomes of oocyte retrievals with and without anesthesia.

Outcome	Anesthesia	No anesthesia	*p*-value
Number of oocytes retrieved	4.6 ± 3.0	2.85 ± 2.8	< 0.01
Mature oocytes rate, %	45.2 ± 40.2	38.7 ± 42.1	0.96
Fertilization rate, % (fertilized/retrieved oocytes)	53.4 ± 34.8	63.7 ± 33.60	0.02
ET^a^ (ET/total cycles, percentage)	87 (67.4%)	76 (65.5%)	0.75
Percentage of patients with good quality embryos	24/39 (61.5%)	9/18 (50%)	0.41
Day of ET, n (%)			< 0.01
Day 3	33/73 (45.2%)	58/78 (74.4%)	
Day 5	40/73 (54.8%)	20/78 (25.6%)	
Ratio of follicles retrieved/expected	0.83 ± 0.8	0.84 ± 0.4	0.96
Pregnancy			
Pregnancy, n (%) per ET	39/87 (44.8%)	20/76 (26.3%)	< 0.01
Abortion, n (%) per pregnancy	11/39 (28.2%)	3/20 (15.0%)	0.26
Ectopic, n (%) per pregnancy	1/39 (2.6%)	0/20 (0%)	0.47
Chemical, n (%) per pregnancy	1/39 (2.6%)	0/20 (0%)	0.47
Delivery, n (%) per pregnancy	26/39 (66.7%)	17/20 (85.0%)	0.13

Data are reported as mean ± standard error of the mean or n (%).

^a^ET, embryo transfer.

When patient data were adjusted for age, BMI, estradiol on triggering day and total gonadotropin dosage, there was no significant difference in the ratio of expected to retrieved oocytes between retrievals with and without anesthesia (0.8 ± 0.4 vs. 0.8 ± 0.8, respectively, *p* = 0.96). Anesthesia parameters are shown in Table 3. A further analysis to examine the possible association between anesthesia parameters and IVF outcomes did not find any associations between desaturation events, lower mean non-invasive blood pressure, duration of anesthesia, propofol dosage and IVF outcomes, such as fertilization rate, embryo quality and pregnancy rate.

**Table 3:** Anesthesia parameters.

Desaturation events (percentage)^a^	15/129 (11.6%)
Mean non-invasive blood pressure (mmHg)	75.59 ± 10.4
< 60 mmHg	5/129 (3.9%)
Duration of anesthesia	16.8 ± 6.9
Longer than 15 min	60/129 (46.5%)
Longer than 30 min	4/129 (3.1%)
Propofol dosage (mg)	263.39 ± 138.73

Data are reported as mean ± standard error of the mean or n (%).

^a^Desaturation event: saturation lower than 90%.

## Discussion

This retrospective cohort study examined the effects of general anesthesia with propofol during oocyte retrieval, on oocyte fertilization, embryo development and implantation. Propofol is widely used for intravenous induction of anesthesia in assisted reproduction. It is highly lipophilic with quick onset and short, predictable duration of action due to its rapid penetration of the blood–brain barrier and distribution to the central nervous system^10^. A steady increase in propofol levels proportional to the total dose administered was found in follicular fluid^11^. Accumulation of propofol in follicular fluid may adversely affect oocyte fertilization and embryonic development. This negative effect may be attributed to the ability of propofol to effect the oocyte-plasma membrane, as significant decreases in the rate of sperm fusion, as well as in pronuclei and polar body formation were observed in mouse oocytes exposed to propofol, compared to controls^5^. Propofol also causes hypotension and rarely, intraoperative desaturation. A prospective study observed higher oxygen consumption rates for human oocytes undergoing normal fertilization compared with nonfertilized or abnormal oocytes^12^. The same study also reported higher oxygen consumption rates among oocytes that generated embryos that implanted, compared with those that did not. Intraoperative desaturation impairs oxygen delivery to oocytes. Decreased oxygen for consumption may be another explanation for sub-optimal fertilization, embryo development and implantation after propofol exposure.

The information reported here suggests that general anesthesia during oocyte retrieval reduces oocyte fertilization rates. Fertilization rate was reported as an indicator of cumulative live birth rate^13^; highlighting the significance of our findings. Potential mechanisms for the negative effect of propofol on oocyte fertilization include direct effects of oolemma perturbation and indirect effects of impaired perfusion to oocytes due to hemodynamic changes. In contrast to the results reported here, a few studies investigated the effect of anesthesia, specifically propofol and reported no effect on IVF outcomes^14^. However, the patients in these studies differed from those in the current investigation. Two studies compared the use of propofol to thiopental and one study compared it to isoflurane^15–17^. A review by Sharma et al., reported inconclusive findings regarding the effect of anesthesia on IVF outcomes, which could be attributed to differences in study designs, drugs and protocols used^18^. The current study compared propofol use to no anesthesia, which enabled us to assess the effect of propofol, more clearly.

A randomized controlled study found no significant differences in fertilization rates between high- and low-dose propofol but the clinical pregnancy rate in the high-dose group was significantly lower^7^.

The lower fertilization rate found in the anesthesia group suggests that it was negatively affected above a threshold level of propofol exposure. In addition, the effect may be all-or-none, rather than dose-dependent.

Another aspect to consider is the use of analgesia. Fentanyl was also administrated to the anesthesia group and may have contributed to the lower fertilization rate. Steady increases in fentanyl concentration in follicular fluid up to 25 min after an IV bolus dose have been reported^19,20^. However, another study reported that fentanyl and remifentanil, do not decrease IVF success rates^21^.

In the current study, patients who were not exposed to anesthesia were slightly older than those who were. They had fewer oocytes expected to be retrieved according to their last ultrasound follicle count, and higher estrogen and FSH levels before triggering. Given these baseline characteristics, this group of patients was expected to have poorer IVF outcomes. Nevertheless, their oocyte fertilization rate was higher. This information may influence patient consultations regarding anesthesia during oocyte retrieval.

We also evaluated the effects of anesthesia-associated parameters, such as desaturation events and anesthesia duration, on IVF outcomes. Desaturation events decrease perfusion to the endometrium and may influence its receptivity to embryo implantation^19,22^. While some studies reported lower implantation rates per transferred embryo and pregnancies per cycle, with lower uterine artery blood flow^22,23^, others did not find differences in implantation and pregnancy rates with low, medium or high uterine artery blood flow^24,25^. We did not find negative effects from desaturation events or longer duration of anesthesia, or lower mean non-invasive blood pressure, on IVF outcomes. However, a trend toward lower pregnancy and delivery rates was observed among patients who underwent anesthesia. The results suggest an additional possible all-or-none effect of anesthesia on embryo implantation and development.

This study was limited by its retrospective nature. Anesthesia administration was not randomized, but based on patient preference. Patients with low follicle counts, expecting short retrieval procedures may be more likely to waive anesthesia. Therefore, the results should be interpreted with this potential selection bias in mind and not extrapolated to the general population undergoing IVF. Furthermore, the possible contribution of fentanyl cannot be dismissed and should be investigated in future studies.

Previous studies examined the effects of other anesthetic or analgesic agents. However, most did not have a control group of patients who were not treated with sedatives, anesthesia or analgesic agents. Therefore, the effect of general anesthesia on IVF outcomes cannot be extrapolated from them. An additional strength of our study is the control group of patients who did not use any type of pain control medication during the retrieval.

A secondary outcome measure was the efficiency of follicle retrieval associated with anesthesia, measured as the ratio of retrieved oocytes to the number recorded at the last ultrasonogram before triggering. We hypothesized that without anesthesia, the operator may be more likely to forgo small follicles due to patient discomfort. Our results showed no difference in the efficiency of small follicle retrieval according to anesthesia use. This may reassure women who do not want anesthesia that this decision will not affect the efficiency of follicle retrieval. We also found that 53% of patients who underwent another IVF cycle chose not to use anesthesia. Given that anesthesia is very accessible and affordable in Israel, we consider this a relatively high percentage of patients waiving anesthesia. This may be due to concerns regarding potential complications of general anesthesia^26^, as well as its potential effects on oocyte quality. On the other hand, the use of anesthesia for oocyte retrieval is still routine practice in IVF. In addition to patient comfort, it is considered necessary for optimal performance of the retrieval and to avoid complications such as hemorrhage or injury to adjacent structures^27^. In our experience, careful patient selection and detailed discussion with patients, enables the physician to perform the procedure accurately and safely without anesthesia, along with good patient satisfaction.

In conclusion, we found that oocytes retrieved under general anesthesia had significantly lower fertilization rates than did those retrieved without anesthesia. This effect on the developmental potential of oocytes may lead to negative IVF outcomes and should be investigated further. However, these results may reassure and even encourage patients who are considering oocyte retrieval without general anesthesia.

## Data Availability

The data that support the findings of this study are available on request from the corresponding author. The data are not publicly available due to privacy or ethical restrictions.
